# Simulation Study on Performance Optimization of Magnetic Nanoparticles DC Thermometry Model

**DOI:** 10.3390/s21072404

**Published:** 2021-03-31

**Authors:** Yapeng Zhang, Shuangbao Ma, Wenzhong Liu

**Affiliations:** 1 School of Mechanical Engineering and Automation, Wuhan Textile University, Wuhan 430073, China; ypzhang@wtu.edu.cn; 2 Hubei Key Laboratory of Digital Textile Equipment, Wuhan Textile University, Wuhan 430073, China; 3 State Key Laboratory of New Textile Materials and Advanced Processing Technologies, Wuhan Textile University, Wuhan 430073, China; 4 School of Artificial Intelligence and Automation, Huazhong University of Science and Technology, Wuhan 430074, China; lwz7410@hust.edu.cn

**Keywords:** magnetic nanoparticles, thermometry, magnetics-based thermometry, magnetizations

## Abstract

Magnetic nanoparticles (MNPs) can work as temperature sensors to realize temperature measurement due to the excellent temperature sensitivity of their magnetization. This paper mainly reports on a performance optimization method of MNPs DC thermometry model. Firstly, by exploring the influencing factors of MNPs magnetization temperature sensitivity, it is found that the optimal excitation of the magnetic field to make the temperature sensitivity of MNPs reach their optimal value is, approximately, inversely proportional to the particle size of MNPs. Then, the temperature sensitivity of MNP magnetization is modulated by adding appropriate DC bias magnetic field in the original triangular wave excitation field, to optimize the original DC thermometry model based on MNP magnetization. The simulation results show that the temperature measurement performance of small-size MNPs can be significantly improved. In short, this paper optimizes the temperature measurement performance of the original DC thermometry model based on MNP magnetization and provides a new application idea for temperature measurement of small-size MNPs.

## 1. Introduction

In nature, both physical and chemical process are closely related to temperature. For example, the temperature of cells in different positions and states is not the same, with some cells showing a highest temperature rise of 5–8 °C relative to 37 °C [1]. In hyperthermia [2], the accurate monitoring and control of the temperature and heat dose of the target area is directly related to the effectiveness of the treatment. Therefore, it is very important to measure the temperature accurately in vivo. Due to the resistance, opacity, safety and comfort of organisms, the traditional electrical, optical and acoustic temperature measurement methods encounter great difficulties in characterizing the in vivo temperatures. Magnetics-based thermometry technologies [3,4,5,6,7], such as magnetization-based magnetic nanoparticle (MNP) thermometry and magnetic resonance (MR) thermometry, can directly measure the internal temperature of the measured object through the surface. In addition, even if it contains ferritin, the organism can still be considered as magnetically transparent without additional magnetic interference. Therefore, magnetics-based thermometry is one of the most promising in vivo temperature measurement methods, which has important research significance and application value in the biomedical field.

MNPs composed of magnetic cores and biocompatible coatings have been widely used in biological and medical fields [8,9,10]. In addition, MNPs are one of the most efficient temperature-to-magnetic conversion media known to date, which can achieve excellent temperature measurement [11,12,13,14]. Magnetic sensors, such as differential coils, can be generally selected to measure the magnetization response signal of MNPs excited by a direct current (DC), or low-frequency alternating current (AC) magnetic field. Then, the relationship between MNP magnetization response and temperature is established to realize the temperature measurement or temperature model establishment. However, the performance of the magnetization-based MNP thermometry is worthy of further optimization. For example, the influence of the temperature sensitivity of MNP magnetization on the measurement performance of MNP thermometry is not considered in detail. In our previous work [15], we found that the temperature sensitivity of MNP magnetization is affected by its working conditions, such as the excitation magnetic field and the size of MNPs. Therefore, adjusting the working conditions of MNPs to optimize their temperature sensitivity may be the key to improving the performance of magnetization-based MNP thermometry. 

Therefore, this paper mainly focuses on the optimization of magnetization-based MNP thermometry by means of simulation. Firstly, the influencing factors of MNP’s magnetization temperature sensitivity are further explored, including excitation magnetic field, MNPs’ size, etc. This gives the regulation law of the temperature sensitivity of MNPs, that is, the optimal excitation magnetic field makes the temperature sensitivity of MNPs reach their optimal value, which is approximately inversely proportional to the particle size of MNPs. Then, based on the regulation of the external magnetic field and the MNPs’ size on the temperature sensitivity of MNPs, the temperature measurement model of MNPs in the low-frequency triangular wave excitation magnetic field was optimized. Through simulations, it is found that by superimposing an appropriate DC bias magnetic field in the low-frequency triangular wave excitation magnetic field, the standard deviation of the temperature measurement error of MNPs with small particle size can be effectively reduced, and temperature measurement performance can be effectively improved. This paper not only further verifies and emphasizes influence factors and laws of temperature sensitivity in MNP magnetization, but also provides ideas for the optimization of magnetization-based MNP thermometry.

## 2. Model and Method

### 2.1. Langevin Model and DC Thermometry Model of MNPs

**Figure 1:**
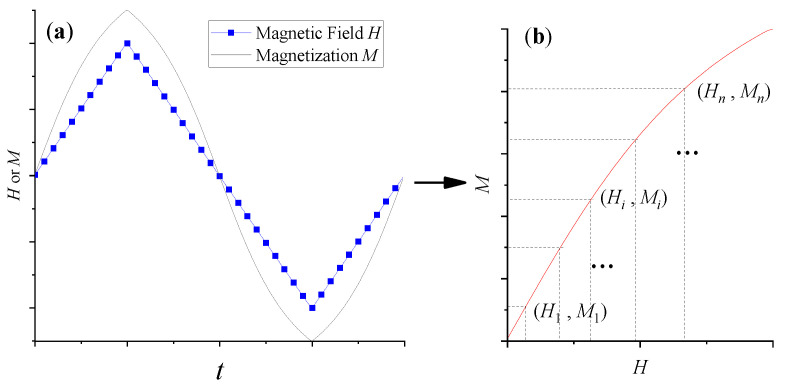
Acquisition, symmetry and discretization of M–H magnetization curve of Magnetic nanoparticles (MNPs) in a low-frequency triangular wave excitation magnetic field. (**a**) The low-frequency triangular wave magnetic field *(H*) and the magnetization (*M*) of MNPs versus time (*t*), respectively. (**b**) The corresponding M-H magnetization curve after quarter period symmetry processing.

According to the classical Langevin equation [16,17], the magnetization response of MNPs in magnetic fields is directly related to temperature; in the same magnetic field, the magnetization response of MNPs shows a downward trend with an increasing temperature. Therefore, the temperature information can be extracted from the magnetization M versus the applied magnetic field H curve (M–H magnetization curve) of MNPs. However, commonly used measuring instruments, such as vibrating sample magnetometer (VSM), or superconducting quantum interferometer (SQUID), are not only poor in real-time, but also costly. Therefore, Zhong et al. [11,18] built a fast, convenient and low-cost MNPs magnetization measurement system to realize fast measurement of temperature information. The measurement system uses a Helmholtz coil or solenoid to provide a DC or low-frequency AC excitation magnetic field, and measures the corresponding magnetization signal of MNPs using differential coils. In the low-frequency excitation AC magnetic field, the effect of relaxation mechanism on the magnetization response of MNPs can be ignored, so the magnetization response of MNPs still follows the Langevin equation. Therefore, a set of (H_*i*_, M_*i*_) data can be obtained by exciting the MNPs sample, using a low-frequency triangular wave magnetic field, and measuring the corresponding magnetization response in real-time. Then, the temperature information can be obtained through the DC thermometry model of MNPs shown in the following, Equation (1) [11].

(1) $$\{\begin{array}{c} M_{1}=x\left(\coth yH_{1}-1/\left(yH_{1}\right)\right)\\ \vdots \\ M_{i}=x\left(\coth yH_{i}-1/\left(yH_{i}\right)\right)\\ \vdots \\ M_{n}=x\left(\coth yH_{n}-1/\left(yH_{n}\right)\right) \end{array},$$

where $x=f_{v}M_{s}$, $y=\frac{M_{s}V}{k_{B}T}$. *f_v_* is the effective volume fraction of MNPs in the suspension. *M_s_* and *V* are the saturation magnetization and volume of the MNP, respectively. *k_B_* is the Boltzmann constant. *T* is absolute temperature in Kelvin. *H_i_* is the discretization of the excited magnetic field *H*, and *M_i_* is the corresponding magnetization of the MNPs sample. In addition, as shown in Figure 1, due to the symmetry of triangular wave, the *M*–*H* magnetization curve of MNPs, excited by a low-frequency triangular wave magnetic field, can be operated symmetrically to improve the performance of this DC thermometry model.

### 2.2. Temperature Sensitivity of the MNP Magnetization

**Figure 2:**
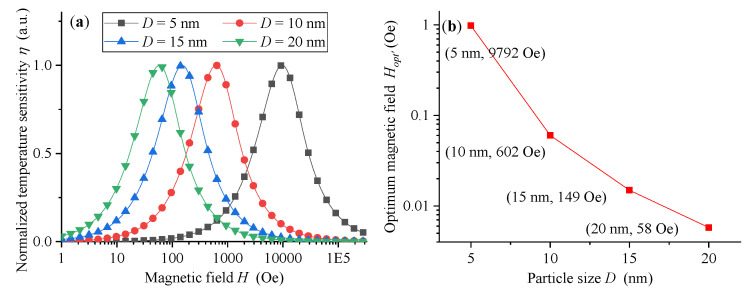
(**a**) The relationship between the temperature sensitivity (η) and static magnetic field (H) for different MNP size (*D*). (**b**) Influence of particle size (*D*) of MNPs on their optimal static magnetic field (H_*opt*_).

MNPs can be used for temperature measurement due to the excellent temperature sensitivity of their magnetization response. The temperature sensitivity of the MNPs, which has already been analyzed in our recent work [15], is critical to the signal to noise ratio (SNR) and the resolution of magnetization-based MNP thermometry

(2) $$\eta =\left|\frac{\partial M}{\partial T}\right|=f_{v}M_{s}\left|\frac{\beta \left({\left(\coth\frac{\beta }{T}\right)}^{2}-1\right)}{T^{2}}-\frac{1}{\beta }\right|,$$

where $\beta =\frac{M_{s}\frac{\pi D^{3}}{6}H}{k_{B}}$ characterizes the effect of particle size *D*, saturation magnetization *M_s_*, and static magnetic field *H*. As pointed out in [15], the temperature sensitivity of magnetization response of MNPs is not only related to particle size and saturation magnetization, but also controlled by the exciting static magnetic field. As shown in Figure 2a, the optimal static magnetic field H_*opt*_, which makes the temperature sensitivity of magnetization response of MNPs reach the maximum value, is not a near-zero magnetic field, and the smaller the particle size D of MNPs is, the larger the optimal static magnetic field H_*opt*_ is. More intuitively, the optimal static magnetic field H_*opt*_ is inversely proportional to the cube of the particle size *D*, as shown in Figure 2b. For example, for MNPs with size of 5 nm, the optimal magnetic field H_*opt*_ is about 9792 Oe, while for 10 nm, H_*opt*_ is only about 602 Oe. Therefore, if the working magnetic field range of MNPs is far away from its optimal magnetic field H_*opt*_, the temperature sensitivity of their magnetization response will be significantly reduced, and the temperature measurement performance of the corresponding magnetization-based MNP thermometry will also be significantly weakened. For example, when the magnetic field increases to about 10,000 Oe, the temperature sensitivity of MNPs with a size of 10 nm almost attenuates to 0; while the MNPs with size of 5 nm are not suitable for temperature measurement applications in magnetic fields less than 1000 Oe. In other words, large-size MNPs are more suitable for temperature measurement in low magnetic fields, and vice versa. Therefore, it is beneficial to improve the temperature measurement performance of magnetization-based MNP thermometry by obtaining the optimal magnetic field value and setting the working magnetic field nearby.

### 2.3. Optimization of DC Thermometry Model of MNPs

Based on the above analysis, it is natural to wonder whether it is possible to adjust the working magnetic field point (range) of MNPs by superimposing an appropriate DC bias magnetic field (*H_dc_*) in the original DC thermometry model of MNPs (which is a common method to improve detection in magnetic devices based on magnetization [19,20]), so as to optimize the temperature measurement performance. Then, the corresponding thermometry model can be modified as follows:

(3) $$\{\begin{array}{c} M_{1}=x\left(\coth y\left(H_{dc}+H_{1}\right)-1/\left(y\left(H_{dc}+H_{1}\right)\right)\right)\\ \vdots \\ M_{i}=x\left(\coth y\left(H_{dc}+H_{i}\right)-1/\left(y\left(H_{dc}+H_{i}\right)\right)\right)\\ \vdots \\ M_{n}=x\left(\coth y\left(H_{dc}+H_{n}\right)-1/\left(y\left(H_{dc}+H_{n}\right)\right)\right) \end{array},$$

**Figure 3:**
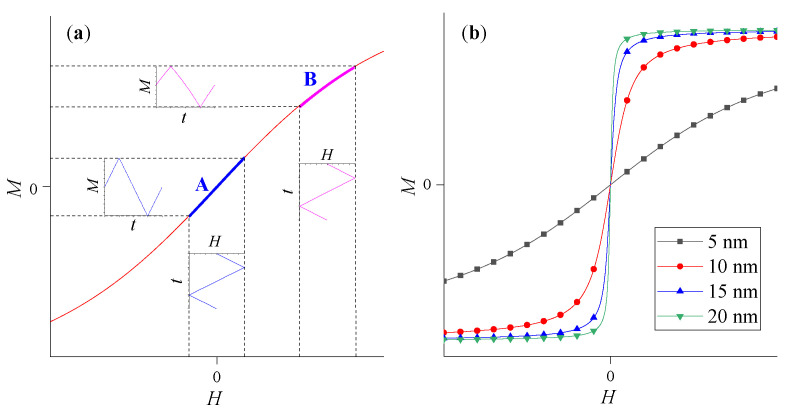
(**a**) M–H magnetization curve of MNP in triangular wave magnetic field with and without DC bias magnetic field. (**b**) M-H magnetization curves of MNP with different sizes.

When the triangular wave magnetic field without DC bias is used, the *M–H* curve is in the linear region near the zero magnetic field. At this time, the corresponding induced magnetization *M* presents the form of non-distorted triangular wave changing with time, as in the waveform marked **A,** shown in Figure 3a. When the DC bias magnetic field is superimposed, the M-H curve enters the nonlinear region and the corresponding induced magnetization *M* presents a slightly distorted triangular waveform with time, as seen in the waveform marked **B**. As shown in Figure 2a, the DC excitation magnetic field which makes the temperature sensitivity of MNPs reach the optimal value, is not a near-zero magnetic field. Therefore, even if the slope of *M–H* curve in **B** range is smaller than that of **A** range, if the superimposed DC bias magnetic field is appropriate, such as MNPs working in **B** range, the magnetization response of MNPs should show better temperature sensitivity than that of **A** range. In addition, the distortion of the *M*–*H* curve can be controlled by adjusting the value of DC bias magnetic field and the amplitude of triangular wave magnetic field. Therefore, by superimposing an appropriate DC bias magnetic field, MNPs can work in the **B** range with better temperature sensitivity, something which may be helpful to improve the temperature measurement performance of the magnetization-based MNP thermometry. Although MNPs can work in the **B** region with better temperature sensitivity by superimposing a DC bias magnetic field, the corresponding induced magnetization M will be reduced even if the amplitude *H_0_* of a triangular wave excitation magnetic field remains the same due to the nonlinearity of **B** region. Therefore, it is necessary to simulate and analyze the influence of DC bias magnetic field *H_dc_*, amplitude *H_0_* of a triangular wave excitation magnetic field, and particle size *D* on the temperature measurement performance of this modified model, to verify the effectiveness of the method.

## 3. Results and Discussion

### 3.1. Simulations of DC Thermometry without H_dc_

From the above analysis, it can be seen that the magnetic field has a decisive influence on the temperature sensitivity of MNP magnetization. Therefore, in temperature measurement applications, MNPs of different particle sizes are suitable for different magnetic field conditions. As shown in Figure 2, the larger the particle size D of MNPs is, the smaller the optimal static magnetic field H_*opt*_ is. Therefore, large-size MNPs seem to be more suitable for DC thermometry without *H_dc_*. Then, the temperature measurement performance of several MNPs with different particle sizes are simulated according to DC thermometry model without *H_dc_*, as shown in Equation (1). In order to reduce the influence of the relaxation mechanism, the frequency of the triangular wave excitation magnetic field is set at a low-frequency of 25 Hz, and a temperature range of 290 K~320 K (near physiological temperature) is simulated and analyzed. In this paper, the Levenberg–Marquardt algorithm [21], a least square estimation method of regression parameters in nonlinear regression, is used to solve the temperature information.

**Figure 4:**
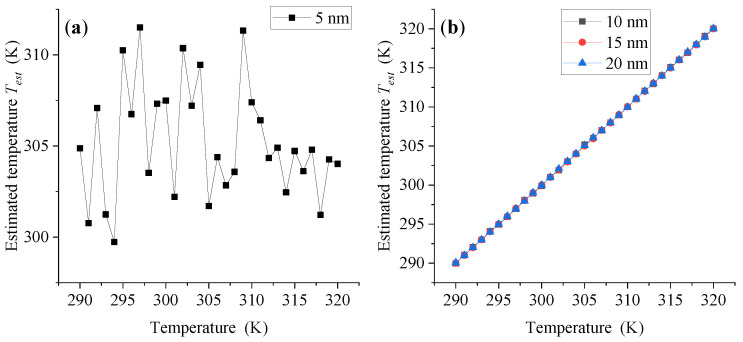
Estimated temperature *T_est_* of MNPs with different sizes at different temperature points when *H*_0_ = 600 Oe.

**Figure 5:**
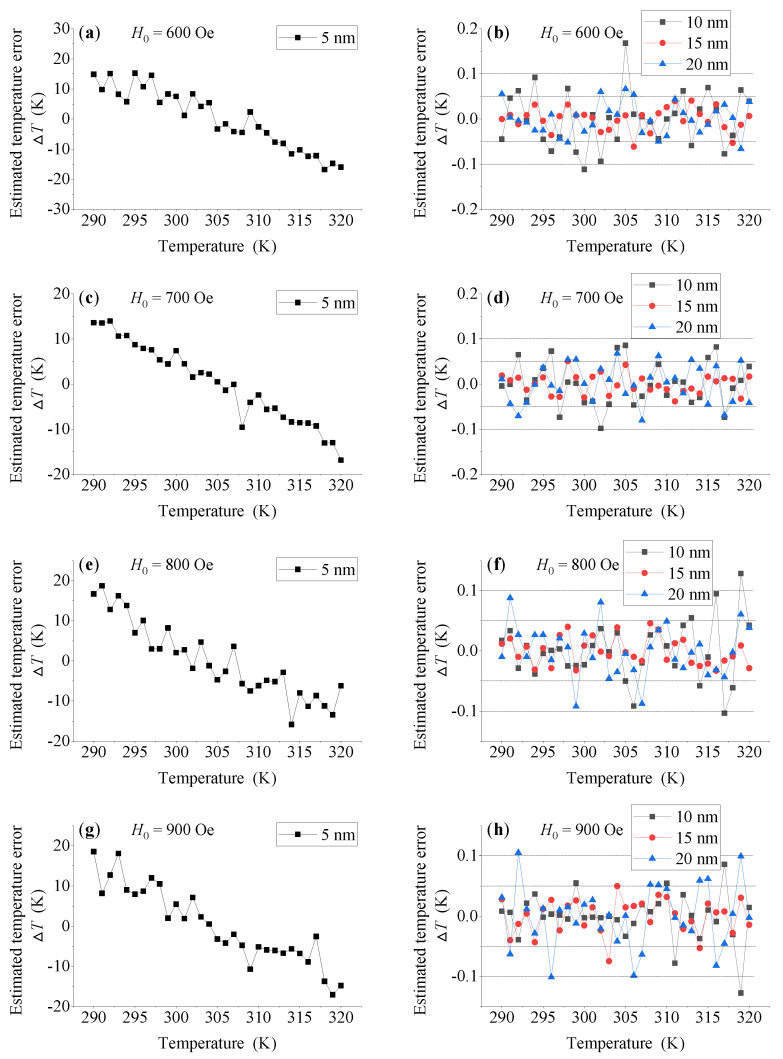
Estimated temperature error ΔT of MNPs with different sizes at different temperature points when *H_0_* = 600 Oe (**a**,**b**), 700 Oe (**c**,**d**), 800 Oe (**e**,**f**) and 900 Oe (**g**,**h**), respectively.

When the amplitude *H_0_* of triangular wave excitation magnetic field is 600 Oe, the estimated temperature *T_est_* and estimated temperature error ΔT at each temperature point are shown in Figure 4 and Figure 5a,b. As we can see, for MNP with size of 5 nm, the estimated temperature *T_est_* fluctuates significantly across the whole measured temperature range, which makes the corresponding estimated temperature error ΔT not only very large, but also vary significantly with the change in measured temperature point. Therefore, the temperature measurement performance of MNP with size of 5 nm cannot meet the needs of temperature measurement. On the contrary, MNPs with a large particle size of 10 nm, 15 nm and 20 nm show better temperature measurement performance, and there is no significant dependence on the measured temperature point. Except for some temperature points, the estimated measurement errors of the three large-size MNPs can be maintained between −0.1 K and 0.1 K in the whole measured temperature range. In addition, when *H_0_* increases to 700 Oe, 800 Oe and 900 Oe, respectively, the temperature measurement performance of MNPs shows a similar change rule as that of *H_0_* = 600 Oe, as shown in Figure 5c–h.

**Figure 6:**
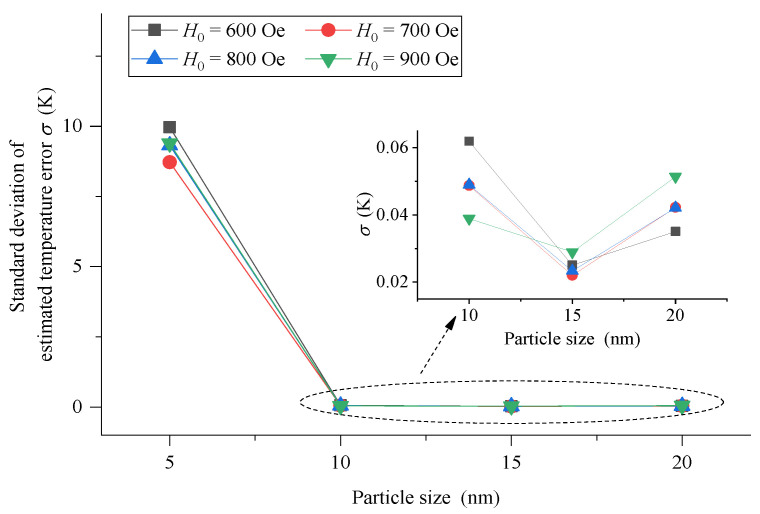
The standard deviation of the estimated measurement error of each temperature point in the whole measured temperature range using MNPs with particle size of 5 nm, 10 nm, 15 nm and 20 nm when *H_0_* = 600 Oe, 700 Oe, 800 Oe and 900 Oe, respectively.

To be more intuitive, the standard deviation of the estimated measurement error of each temperature point in the whole measured temperature range is calculated to comprehensively characterize the temperature measurement performance for MNPs with different particle sizes, as shown in Figure 6. The results show that the standard deviation of 5 nm MNPs is 8 K–10 K, which is not suitable for temperature measurement because of its poor temperature measurement performance. However, with the increase in the particle size of MNPs, the temperature measurement performance is greatly improved. When MNPs with a size of 10 nm, 15 nm and 20 nm are used, the standard deviation is maintained within 0.06 K, which is very excellent. Therefore, high-precision temperature measurement can be realized using large-size MNPs.

**Figure 7:**
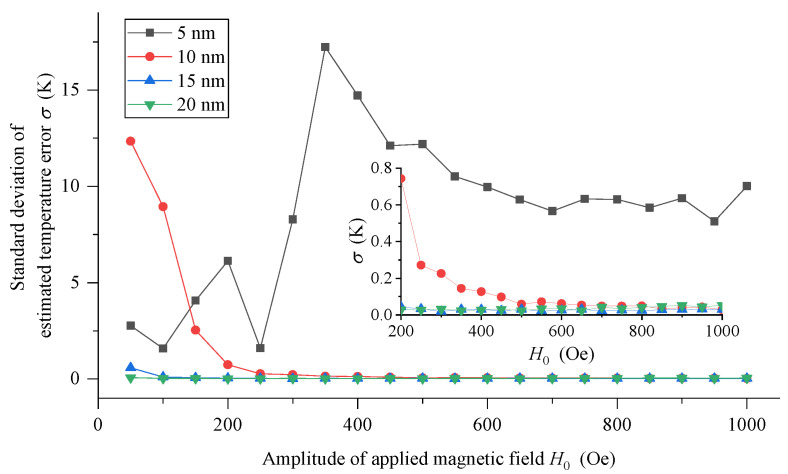
The standard deviation of estimated measurement error of MNPs with different particle sizes with the changing of the amplitude *H_0_*.

Furthermore, the relationship between the standard deviation and the amplitude *H_0_* of the triangular wave magnetic field is given, as shown in Figure 7. The temperature measurement performance of 5 nm MNPs is the worst. The standard deviation is not only very large (several K, or even greater), but also varies irregularly with the increase in amplitude *H_0_*. Therefore, 5 nm MNPs are not suitable for the DC thermometry model. On the contrary, when MNPs of 10 nm, 15 nm and 20 nm are used, the standard deviation obtained basically shows a trend of becoming better with the increasing amplitude *H_0_*. In addition, the larger the particle size of MNPs is, the smaller the requirement of amplitude *H_0_* is, and an excellent temperature measurement performance can be obtained in the triangular wave excitation magnetic field with smaller amplitude. For example, for 10 nm MNPs, when the amplitude *H_0_* is less than 200 Oe, the standard deviation is greater than 2 K, and the temperature measurement performance is not good. While when the amplitude *H*_0_ is greater than 200 Oe, the standard deviation will be reduced to less than 1 K; when the amplitude *H*_0_ is greater than 450 Oe, the standard deviation will be further reduced to less than 0.1 K. For 15 nm MNPs, the standard deviation is always less than 1 K; when the amplitude *H_0_* is greater than 150 Oe, the standard deviation will be further reduced to less than 0.1 K. For larger MNPs of 20 nm, the standard deviation is always within 0.1 K, and the dependence on amplitude *H_0_* is not obvious, and excellent temperature measurement performance can be obtained in the range of the studied excitation magnetic field amplitude.

### 3.2. Simulations of DC Thermometry with H_dc_

As shown in Figure 7, when there is no bias magnetic field, the larger the amplitude *H_0_* is; the smaller the standard deviation is, then, the better the temperature measurement performance is. Therefore, we have further focused on the influence of DC bias magnetic fields on the temperature measurement performance of DC thermometry models when the amplitude *H_0_* is large.

**Figure 8:**
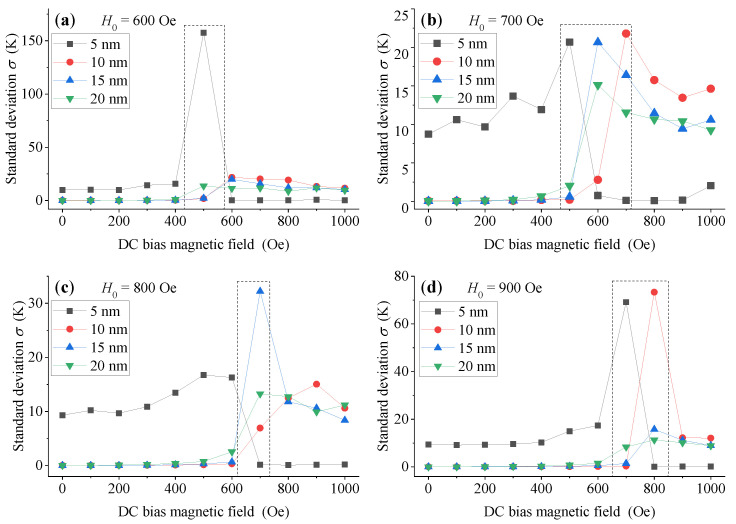
The standard deviation of MNPs with different particle sizes with different DC bias field *H_dc_* when the amplitude *H*_*0*_ of triangular wave excitation magnetic field is (**a**) 600 Oe, (**b**) 700 Oe, (**c**) 800 Oe and (**d**) 900 Oe, respectively.

As shown in Figure 8, except for a few abrupt abnormal points (in the dotted line box), MNPs with large-size (10 nm, 15 nm and 20 nm) show good temperature measurement performance when the DC bias magnetic field *H_dc_* is small, while small-size (5 nm) MNPs show good temperature measurement performance when *H_dc_* is large. Moreover, when there is no DC bias magnetic field, the temperature measurement performance of 5 nm MNPs is very poor (the corresponding standard deviation can reach to several K, even more than 10 K), as shown in Figure 7 above. Therefore, we can conclude that adding appropriate DC bias magnetic field *H_dc_* in the low-frequency triangular wave excitation magnetic field can significantly improve the temperature measurement performance of DC thermometry model, with a small particle size MNPs of 5nm. For large-size MNPs (10 nm, 15 nm and 20 nm), we can obtain an excellent temperature measurement performance by utilizing a low DC bias magnetic field *H_dc_*. However, if we increase the DC bias magnetic field *H_dc_*, corresponding temperature measurement performance will degrade due to suppression of the temperature sensitivity of MNPs. Thus, the superposition of DC bias magnetic field makes large-size MNPs work in a region far away from the optimal range of their temperature sensitivity, which worsens their temperature measurement performance.

Furthermore, we can define the conversion magnetic field; when the DC bias magnetic field *H_dc_* reaches this value, the temperature measurement performance of the DC thermometry model of MNPs changes significantly, as shown in the dotted box in Figure 8. Therefore, in order to obtain an excellent temperature measurement performance using small-size MNPs, the applied DC bias magnetic field *H_dc_* should be larger than the corresponding conversion magnetic field. On the contrary, when using large size MNPs, the applied DC bias magnetic field *H_dc_* should be smaller than the corresponding conversion magnetic field.

**Figure 9:**
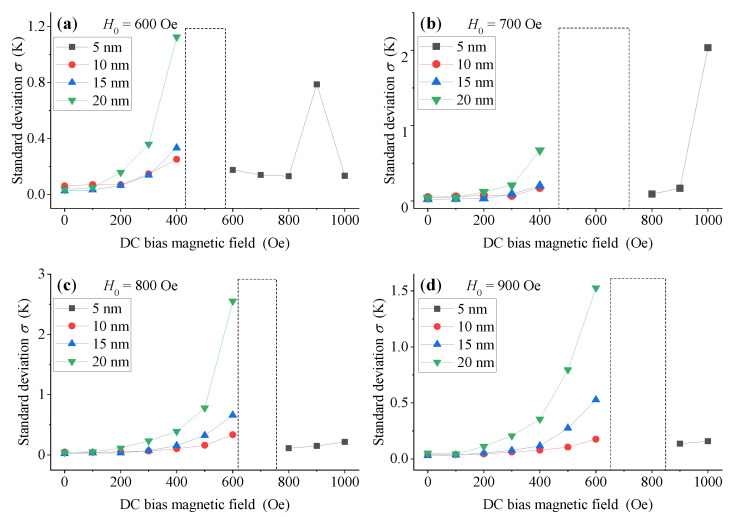
The standard deviation of the large-size MNPs (10 nm, 15 nm and 20 nm) when *H_dc_* is smaller than the conversion magnetic field and the standard deviation of the small-size MNPs (5 nm) when *H_dc_* is larger than the conversion magnetic field.

We see, therefore, that abnormal values near the conversion magnetic field are hidden, and the standard deviation of large-size MNPs (when *H_dc_* is smaller than the conversion magnetic field, as well as the standard deviation of small-size MNPs when *H_dc_* is larger than the conversion magnetic field) are mainly focused on, as shown in Figure 9. As we can see, even when *H_dc_* is smaller than the conversion magnetic field, the superposition of the DC bias magnetic field in the low-frequency triangular wave excitation magnetic field will worsen the temperature measurement performance of large-size MNPs. Conversely, the temperature measurement performance of small-size MNPs will be significantly improved by superposing a DC bias magnetic field larger than the conversion magnetic field.

**Figure 10:**
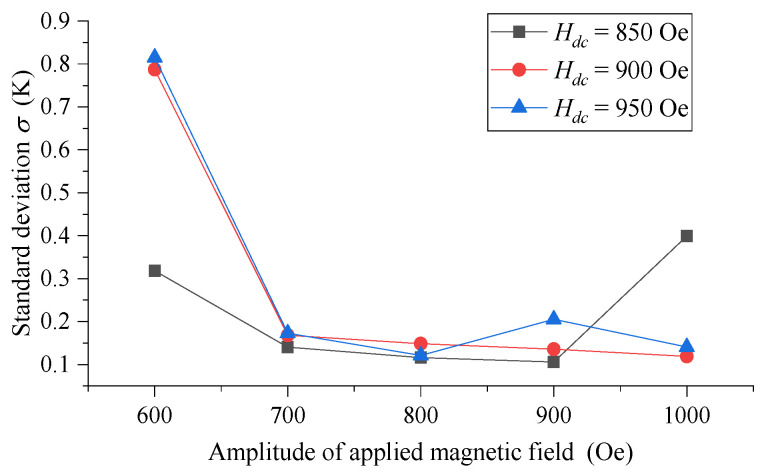
The standard deviation of 5 nm MNPs with the changing amplitude of triangular wave excitation magnetic field in different DC bias magnetic field *H_dc_*.

The standard deviation of small-size MNPs (5 nm) when *H_dc_* is large is given in Figure 10. It can be seen that the temperature measurement performance of 5 nm MNPs has been significantly improved when compared with that without *H_dc_*. Except for a few points, the standard deviation of 5 nm MNPs is overall better than 0.5 K, and the optimal value is between 0.10 K and 0.15 K. Therefore, we can improve the temperature measurement performance of small-size MNPs (5 nm) significantly by superposing appropriate DC bias magnetic field into the original low-frequency triangular wave magnetic field.

### 3.3. Discussion

In this paper, we optimize the temperature measurement performance of the MNP DC thermometry model by means of simulation, using the modulation law of external magnetic field and particle size on the temperature sensitivity of the induced magnetization of MNPs, but there are still some shortcomings and problems to be solved. In our simulations, we assume that the MNPs are of uniform size. However, the actual MNPs reagent will present a particle size distribution in the form of lognormal function [22], which will certainly affect the corresponding simulation law of the temperature sensitivity of MNPs and the optimization results of the MNP DC thermometry model. We preliminarily discussed the effect of particle size distribution on the temperature sensitivity of MNPs, and found that the increase in particle size distribution will cause the optimal temperature sensitivity value and the corresponding optimal static magnetic field to show a decreasing trend. The corresponding content is not presented in this article, due to the need for further research. Moreover, saturation magnetization M_*s*_ of MNP is also related to temperature, which can be described by the Bloch’ law [23], and the change in saturation magnetization with temperature will also affect the simulation results presented in this manuscript. Therefore, this also needs further research. In addition, the causes of the abnormal points in Figure 8 and the so-called conversion magnetic field also need to be further studied.

## 4. Conclusions

This paper further analyzed the temperature sensitivity of the induced magnetization of MNPs, and points out that the temperature sensitivity of the MNP magnetization is the key to the temperature measurement performance of magnetization-based MNP thermometry. Through simulation, it was found that the optimal excitation of magnetic fields to make the temperature sensitivity of MNPs reach their optimal value, is approximately inversely proportional to the particle size of MNPs. Therefore, MNPs with a small particle size, concentrated particle size distribution, and high saturation magnetization are more suitable for temperature measurement applications in a high magnetic field, and vice versa. Then, the DC thermometry model of magnetization-based MNP thermometry was optimized and regulated by adding an appropriate DC bias magnetic field in the triangular wave excitation magnetic field. The simulation results showed that the standard deviation of estimated temperature error in small-size MNPs can be decreased from several K or even more than 20 K to approximately 0.15 K by adding an appropriate DC bias magnetic field, which provides a new idea for the application of temperature measurement of small-size MNPs.

## Data Availability

All data generated or appeared in this study are available upon request by contact with the corresponding author.

## References

[1] Yang J.M., Yang H., Lin L. (2011). Quantum dot nano thermometers reveal heterogeneous local thermogenesis in living cells. ACS Nano.

[2] Adibzadeh F., Sümser K., Curto S., Yeo D.T.B., Paulides M.M. (2020). Systematic review of pre-clinical and clinical devices for magnetic resonance-guided radiofrequency hyperthermia. Int. J. Hyperth..

[3] Pi Ol R., Brites C.D.S., Bustamante R., Martínez A., Silva N.J.O., Murillo J.L., Cases R., Carrey J., Estepa C., Sosa C. (2015). Joining Time-Resolved Thermometry and Magnetic-Induced Heating in a Single Nanoparticle Unveils Intriguing Thermal Properties. ACS Nano.

[4] Winter L., Oberacker E., Paul K., Ji Y., Oezerdem C., Ghadjar P., Thieme A., Budach V., Wust P., Niendorf T. (2016). Magnetic resonance thermometry: Methodology, pitfalls and practical solutions. Int. J. Hyperth..

[5] Baron P., Deckers R., Knuttel F.M., Bartels L.W. (2015). T1 and T2 temperature dependence of female human breast adipose tissue at 1.5 T: Groundwork for monitoring thermal therapies in the breast. NMR Biomed..

[6] Cline H.E., Hynynen K., Watkins R.D., Adams W.J., Schenck J.F., Ettinger R.H., Freund W.R., Vetro J.P., Jolesz F.A. (1995). Focused US system for MR imaging-guided tumor ablation. Radiology.

[7] Hynynen K., Darkazanli A., Unger E., Schenck J.F. (1993). MRI-guided noninvasive ultrasound surgery. Med. Phys..

[8] Zhou Y., Yan D., Yuan S., Chen Y., Fletcher E.E., Shi H., Han B. (2017). Selective binding, magnetic separation and purification of histidine-tagged protein using biopolymer magnetic core-shell nanoparticles. Protn. Exp. Purif..

[9] Tietze R., Zaloga J., Unterweger H., Lyer S., Alexiou C. (2015). Magnetic Nanoparticle-based Drug Delivery for Cancer Therapy. Biochem. Biophy. Res. Commun..

[10] Crespo P., Patricia D.L.P., Marín P., Multigner M., María Alonso J., Rivero G., Yndurain F., María González-Calbet J., Hernando A. (2013). Magnetism in nanoparticles: Tuning properties with coatings. J. Phys. Condens. Matter.

[11] Zhong J., Liu W., Jiang L., Yang M., Morais P.C. (2014). Real-time magnetic nanothermometry: The use of magnetization of magnetic nanoparticles assessed under low frequency triangle-wave magnetic fields. Rev. Sci. Instrum..

[12] Perreard I.M., Reeves D.B., Zhang X., Kuehlert E., Forauer E.R., Weaver J.B. (2014). Temperature of the magnetic nanoparticle microenvironment: Estimation from relaxation times. Phys. Med. Biol..

[13] Weaver J.B., Rauwerdink A.M., Hansen E.W. (2009). Magnetic nanoparticle temperature estimation. Med. Phys..

[14] Rauwerdink A.M., Hansen E.W., Weaver J.B. (2009). Nanoparticle temperature estimation in combined ac and dc magnetic fields. Phys. Med. Biol..

[15] Zhang Y., Guo S., Zhang P., Zhong J., Liu W. (2020). Iron oxide magnetic nanoparticles based low-field MR thermometry. Nanotechnology.

[16] Chikazumi S., Taketomi S., Ukita M., Mizukami M., Miyajima H., Setogawa M., Kurihara Y. (1987). Physics of magnetic fluids. J. Magn. Magn. Mater..

[17] Kaiser R. (1970). Magnetic Properties of Stable Dispersions of Subdomain Magnetite Particles. J. Appl. Phys..

[18] He L., Liu W., Xie Q., Pi S., Morais P.C. (2016). A fast and remote magnetonanothermometry for a liquid environment. Meas. Sci. Technol..

[19] Hauser M., Kraus L., Ripka P. (2001). Giant magnetoimpedance sensors. IEEE Instrum. Meas. Mag..

[20] Herrero-Gomez C., Marin P., Hernando A. (2013). Bias free magnetomechanical coupling on magnetic microwires for sensing applications. Appl. Phys. Lett..

[21] Moré J.J. (1978). The Levenberg-Marquardt Algorithm: Implementation and Theory. Numerical Analysis.

[22] Lak A., Ludwig F., Scholtyssek J.M., Dieckhoff J., Fiege K., Schilling M. (2013). Size Distribution and Magnetization Optimization of Single-Core Iron Oxide Nanoparticles by Exploiting Design of Experiment Methodology. IEEE Trans. Magn..

[23] Della Torre E., Bennett L.H., Watson R.E. (2005). Extension of the BLOCH T(3/2) law to magnetic nanostructures: Bose-Einstein condensation. Phys. Rev. Lett..

